# Characterization of *Danio rerio* Mn^2+^-Dependent ADP-Ribose/CDP-Alcohol Diphosphatase, the Structural Prototype of the ADPRibase-Mn-Like Protein Family

**DOI:** 10.1371/journal.pone.0042249

**Published:** 2012-07-27

**Authors:** Joaquim Rui Rodrigues, Ascensión Fernández, José Canales, Alicia Cabezas, João Meireles Ribeiro, María Jesús Costas, José Carlos Cameselle

**Affiliations:** 1 Grupo de Enzimología, Departamento de Bioquímica y Biología Molecular y Genética, Facultad de Medicina, Universidad de Extremadura, Badajoz, Spain; 2 Escola Superior de Tecnologia e Gestão, Instituto Politécnico de Leiria, Leiria, Portugal; Institute of Enzymology of the Hungarian Academy of Science, Hungary

## Abstract

The ADPRibase-Mn-like protein family, that belongs to the metallo-dependent phosphatase superfamily, has different functional and structural prototypes. The functional one is the Mn^2+^-dependent ADP-ribose/CDP-alcohol diphosphatase from *Rattus norvegicus*, which is essentially inactive with Mg^2+^ and active with low micromolar Mn^2+^ in the hydrolysis of the phosphoanhydride linkages of ADP-ribose, CDP-alcohols and cyclic ADP-ribose (cADPR) in order of decreasing efficiency. The structural prototype of the family is a *Danio rerio* protein with a known crystallographic structure but functionally uncharacterized. To estimate the structure-function correlation with the same protein, the activities of zebrafish ADPRibase-Mn were studied. Differences between zebrafish and rat enzymes are highlighted. The former showed a complex activity dependence on Mn^2+^, significant (≈25%) Mg^2+^-dependent activity, but was almost inactive on cADPR (150-fold less efficient than the rat counterpart). The low cADPR hydrolase activity agreed with the zebrafish genome lacking genes coding for proteins with significant homology with cADPR-forming enzymes. Substrate-docking to zebrafish wild-type protein, and characterization of the ADPRibase-Mn H97A mutant pointed to a role of His-97 in catalysis by orientation, and to a bidentate water bridging the dinuclear metal center as the potential nucleophile. Finally, three structural elements that delimit the active site entrance in the zebrafish protein were identified as unique to the ADPRibase-Mn-like family within the metallo-dependent phosphatase superfamily.

## Introduction

The structure of the binuclear metallophosphoesterases or metallo-dependent phosphatases (MDP) superfamily (SCOP accession ID SSF56300) contains a dimetal center with diverse ion pairs or combinations; a βαβαβ secondary structure signature within a four-layered fold with two β-sheets flanked by α-helices (α/β/β/α fold); and a disperse sequence signature that includes, in five conserved regions, the amino acids coordinated with the metal ions: DX[H/X]-(X)n-GDXX[D/X]-(X)n-GNH[D/E]-(X)n-[G/X]H-(X)n-GHX[H/X] [1]–[5]. The Mn^2+^-dependent ADP-ribose/CDP-alcohol diphosphatase (ADPRibase-Mn; EC 3.6.1.53) has been recently classified within the MDP superfamily, where ADPRibase-Mn-like proteins form a unique SCOP family [6]. The structural prototype of this family is a zebrafish protein, encoded by *Danio rerio* gene Zgc:64213. It was chosen a few years ago for crystallographic structure determination [7], [8] as a hypothetical protein then lacking structurally- and biochemically-studied close homologues. The structure of this protein, complexed with P_i_ and four Zn^2+^ ions (two outside the dimetal center), is recorded in Protein Data Bank (PDB) under ID 2nxf [9]. However, it remains otherwise uncharacterized. In fact, the only family member that has been enzymatically studied is rat ADPRibase-Mn [10]–[12]. It was first found to act on ADP-ribose (its best substrate), CDP-choline, CDP-glycerol, CDP-ethanolamine and ADP, with a marked dependency on low micromolar concentrations of Mn^2+^ which cannot be substituted by Mg^2+^ even at millimolar concentrations [10], [11]. More recently, rat ADPRibase-Mn has been unexpectedly found to be also active in vitro towards the phosphoanhydride linkage of cyclic ADP-ribose (cADPR) [12], which is resistant even to broad-specificity phosphodiesterases [13], [14]. In this regard, it is the only known alternative to the enzymatic turnover of this universal calcium regulator by the same proteins that form it from NAD: the mammalian membrane-bound NAD-glycohydrolases (NADases) CD38 [13], [15], BST-1/CD157 [16], and mitochondrial NADase [17]. ADPRibase-Mn converts cADPR to *N* ^1^-(5-phosphoribosyl)-AMP [12], an outcome totally different to the hydrolysis of cADPR by the above mentioned NADases that yield ADP-ribose.

ADPRibase-Mn may have a function in vertebrate immune systems. The gene *2310004I24Rik* that encodes the mouse orthologue, is defined as an ‘immune gene’, i.e. one preferentially expressed in immune versus non-immune cells and tissues [18]. Also, rat ADPRibase-Mn mRNAs are more abundant, and ADPRibase-Mn enzyme activity is higher in thymus and spleen (particularly so in splenocytes, which include spleen immunocytes) than in non-immune rat tissues [11]. In agreement with these results, there is a restriction in the taxonomic distribution of ADPRibase-Mn orthologues. While MDP proteins are phylogenetically widespread, the ADPRibase-Mn-like family is restricted, among pluricellular eukaryotes, to vertebrates and higher plants, not being present in invertebrates. Significant ADPRibase-Mn relatives are also absent from most unicellular eukaryotes, including yeasts [11].

The possible immune role of ADPRibase-Mn is unknown. ADP-ribose acts as a second messenger in immune cells by opening TRPM2 ion channels that participate in Ca2+-mediated cell death or leukocyte trafficking [19], [20]. cADPR behaves also as a conditional TRPM2 (co)activator although it seems unclear that it acts directly on the channel protein [21], [22]. Thus one can think that ADPRibase-Mn is perhaps involved in the CD38 (and related enzymes) network with a role in the turnover of ADP-ribose and cADPR, and in the termination of their (in)direct effects on TRPM2 channels in immune cells. Such a ‘signaling’ role would agree with the general role attributed to the greater part of the mouse immune genes showing the expression profiles most similar to that of 2310004I24Rik [11], [18].

To understand the significance of the restricted taxonomic distribution of the ADPRibase-Mn-like family, functional and structural studies of proteins from diverse origins, among other things, are needed. Also, to obtain firm correlation between structure and function, it is necessary to have both aspects studied by experiments performed on the same protein. So far, the ADPRibase-Mn prototypes of structure and function correspond to different proteins, one from zebrafish, the other from the rat. To fill this gap, we present here the functional characterization of zebrafish ADPRibase-Mn. In addition, differences between rodent and fish ADPRibase-Mn, concerning their substrate specificities and their responses to Mn^2+^ and Mg^2+^, are highlighted. A novel activity of ADPRibase-Mn on 2′,3′-cAMP was found. This activity, contrary to other MDPs catalyzing also this reaction, and to the other activities of ADPRibase-Mn itself, was independent on the histidine residue of the GNH[D/E] motif, revealing that this residue affects differentially the activities of ADPRibase-Mn on different substrates. Finally, we are also defining which are the unique structural elements of the ADPRibase-Mn-like family within the MDP superfamily.

## Materials and Methods

### Chemicals and Biochemicals

The sources of most of the products used, including cADPR purified from the commercial preparation, were as described elsewhere [11], [12]. 2′,3′-cAMP and 3′,5′-cAMP were from Sigma-Aldrich. AcTEV™, an enhanced form of tobacco etch virus (TEV) protease, was from Invitrogen.

### Expression and Purification of Zebrafish Recombinant ADPRibase-Mn

Plasmid pVP16-2nxf was obtained from the Protein Initiative Materials Repository (currently at http://psimr.asu.edu/) where it is deposited under ID DrCD00084013. It contains an 8-His tag, followed by maltose-binding protein (MBP), a TEV protease site (*tev*) and the ORF of zebrafish ADPRibase-Mn (GenBank accession No. BC054642 region 60–1028) with a Ser codon substituting for the N-terminal Met one. The *tev*-ADPRibase-Mn sequence was confirmed by re-sequencing from both ends (MBP and pQE forward and reverse primers). The TEV site is located such that the proteolytic cut generates the native ADPRibase-Mn starting with Ser. The plasmid was expressed in 200-ml cultures of transformed BL21 cells with IPTG induction for 2 h at 30°C. The fusion protein 8-His-MBP-*tev*-ADPRibase-Mn was purified from bacterial lysates by immobilized metal affinity chromatography (IMAC) eluted with imidazole, cut with AcTEV protease, and chromatographed again in the IMAC column. ADPRibase-Mn eluted at high purity ahead of the uncut fusion protein. Its identity was confirmed by MALDI-TOF-MS analysis of tryptic digests (Unidad de Proteómica, Universidad Complutense y Parque Científico de Madrid, Spain) which yielded a peptide mass fingerprint covering 69% of the ADPRibase-Mn sequence with high confidence, including the expected 27-mer N-terminal peptide starting with serine, which is confirmatory of the TEV cut.

### Construction of Point Mutant H97A-ADPRibase-Mn

Mutated plasmid pVP16-H97A-2nxf was constructed from pVP16-2nxf according to the QuikChange® mutagenesis procedure (Stratagene), using GGGAATGCCGAGTTCTACAACTTCAGTCGACCATCGCTGCTCTCC and its reverse complement as mutagenic oligonucleotides. PfuUltra™ High Fidelity DNA Polymerase (Stratagene) was used to synthesize mutated chains, and DpnI (New England Biolabs) to degrade the methylated wild-type templates. The correct generation of the mutated ORF in the absence of undesired changes was confirmed by double-strand sequencing. The expression and purification of H97A-ADPRibase-Mn was performed like the wild-type protein.

### Enzyme Assays and Kinetic Studies

Unless otherwise stated, enzyme assays were carried out by measuring the phosphate liberated from reaction products in the presence of alkaline phosphatase, as described earlier [11], [12]. The standard reaction mixtures contained 50 mM Tris-HCl, pH 7.5, 0.1 mg/ml bovine serum albumin, 5 mM MnCl_2_ and 500 µM substrate. In some experiments, the formation of nucleotidic product in the absence of alkaline phosphatase was followed by HPLC (see below).

**Figure 1:**
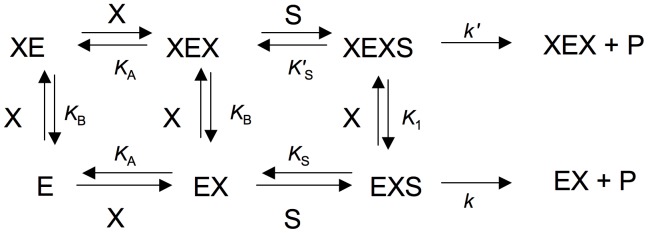
Reaction model including two metal (X) binding sites affecting the kinetics of zebrafish ADPRibase-Mn. Model assumptions are as follows. (i) X can bind to the enzyme (E) at two independent sites with dissociation constants *K*_A_ (site A) and *K*_B_ (site B) to give complex EX and XE, respectively; binding to either site does not affect binding to the other. (ii) On site A, X acts as an essential activator, required for substrate binding. (iii) On site B, X behaves as a general modifier that can increase or decrease the reaction rate depending on other conditions, e.g. the particular substrate, by modifying *K*_S_ (to *K′*_S_) and/or *k* to *k*′ (i.e. *V* to *V′*). (iv) Binding of X to free S is negligible. From this model, kinetic equation I was derived and fit to results from experiments in which the added metal was Mn^2+^ (Fig. 3). As discussed in the text, in these experiments it was assumed that E is an ADPRibase-Mn form with one metal ion (Fe) strongly bound in the dimetalic center. Site A corresponds to the other position of the dimetalic center, and site B would be located outside the active center.

To estimate substrate saturation curves, the Michaelis-Menten equation was fit to data obtained by measuring initial rates at fixed Mn^2+^ and varying substrate concentrations. The catalytic efficiencies or specificity constants (*k*_cat_/*K*_M_) of ADPRibase-Mn with different substrates were determined either from saturation curves or from initial rates measured at substrate concentration at least 8-fold below the *K*_M_ value, when the enzyme is largely unbound to substrate and *k*_cat_/*K*_M_ = *v*/(*E S*), *E* being the total enzyme concentration [23]. To account for the different kinetic responses of zebrafish ADPRibase-Mn reactions to changes of Mn^2+^ concentration with different substrates, the reaction model shown in Fig. 1, inspired by [24], [25], was used. In this case, the corresponding rate equation for the initial velocity, *v*, was derived following the general rules given by Segel for writing velocity equations for rapid equilibrium systems [26]

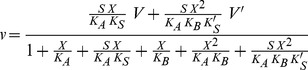
(I)where *S* and *X* stand respectively for total substrate and metal concentrations, *V* = *k E*, *V′* = *k′ E* (*E* being the total enzyme concentration), and all the constants being as defined in Fig. 1. The kinetic equation was fit to experimental data by least squares with the Solver tool of the Microsoft® Excel 2004 program for the Macintosh.

To analyze the results of experiments with two mixed alternative substrates, it was taken in account that each substrate acts as a competitive inhibitor with respect to the other, and that, if product formation from only one of them is measured, the ratio of initial velocity *a* in the presence versus absence of the second substrate is given by
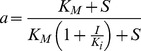
(II)which is deduced [26] from the classical equation for competitive inhibition. *S* and *I* represent the concentrations of the two alternative substrates, *K*_M_ is the Michaelis-Menten constant value for the first substrate (the one yielding the product measured), and *K*_i_ is the inhibition constant of substrate *I*, which equals its own *K*_M_ as substrate [26].

### HPLC Analyses

Nucleotidic products of ADPRibase-Mn were analysed by ion-pair reverse-phase HPLC in a 15 cm×0.4 cm octadecylsilica column (Kromasil 100; Teknokroma, Sant Cugat del Vallés, Barcelona, Spain) with a 1 cm×0.4 cm pre-column of the same material, using a Hewlett–Packard HP1100 chromatograph. Chromatographic conditions for analyses of the reaction products of ADP-ribose, CDP-alcohols and ADP were as described in [11] and those for cADPR in [12]. For the analysis of 2′,3′-cAMP reaction products, two elution buffers were used: buffer A comprised 5 mM sodium phosphate, pH 7.0, 20 mM tetrabutylammonium and 20% (v/v) methanol; buffer B was the same as buffer A, but contained 100 mM sodium phosphate. Before each run, the column was equilibrated in buffer A. Samples (20 µl) of reaction mixture were injected into the chromatograph, and the elution was accomplished at a 1 ml/min flow rate with a linear 10 min 5–100 mM phosphate gradient. The eluates were monitored at 260 nm.

### Docking Simulations

For the docking simulations, the Se atoms of selenomethionine residues of zebrafish ADPRibase-Mn (PDB ID 2nxf) were substituted by S atoms and eight residues which are unresolved in the crystallographic structure (Nos. 1, 2 and 117–122), and are located far apart from the active site, were added with Modeller [27]. Two metal ions present in the dimetal center and a water molecule bound to them were retained for the docking computations. The program Reduce [28] was used to assign the protonation state of histidine side chains and to add hydrogen atoms to the protein, while WhatIf (http://swift.cmbi.ru.nl/servers/html) was used to add hydrogen atoms to the single water molecule. ADT [29] was used to assign atom types and Kollman partial charges to be used with Autodock. The metal ions were modeled as Zn^2+^ ions. 3D models of substrates were generated with Marvin (ChemAxon, Budapest, Hungary; http://www.chemaxon.com). ADT was used to strip off non-polar hydrogen atoms, to compute Gasteiger partial charges and to define the active torsions. The docking computations were carried out with Autodock [30], as described previously [11], [12], except that the number of independent runs was increased so as to have at least 100 poses near the dimetal center for each substrate tested. Cluster analysis of the poses in the binding pocket was performed using the Jarvis-Patrick algorithm [31], as implemented in the g_cluster tool [32], with a cutoff of 2 Å for finding nearest neighbors.

## Results and Discussion

### Zebrafish ADPRibase-Mn Specificity: Preference for ADP-ribose/CDP-alcohols, Negligible cADPR Phosphohydrolase Activity, Significance of the Activity on 2′,3′-cAMP, and Effect of the H97A Mutation

To study the substrate preferences of zebrafish ADPRibase-Mn, the known substrates of the rat enzyme (ADP-ribose, CDP-alcohols, ADP and cADPR) and a selection of non-substrate compounds were used. In addition, and as a novelty with respect to previous studies performed with the rat enzyme, the activity on cyclic 3′,5′- and 2′,3′-phosphodiester nucleotides was also tested. This was decided after learning that the histidine residue equivalent to zebrafish ADPRibase-Mn His-97, the one included in conserved GNH[D/E] motif of the disperse sequence signature of MDPs, is a determinant of 2′,3′-cyclic nucleotide phosphodiesterases in certain proteins of this superfamily [33]. This residue, although not bound to the metal ions of the dinuclear center of either zebrafish ADPRibase-Mn [7], [9] or other MDPs, is known to be important for catalysis [33]–[38]. Therefore, a point mutant H97A-ADPRibase-Mn was also studied in several aspects.

**Figure 2:**
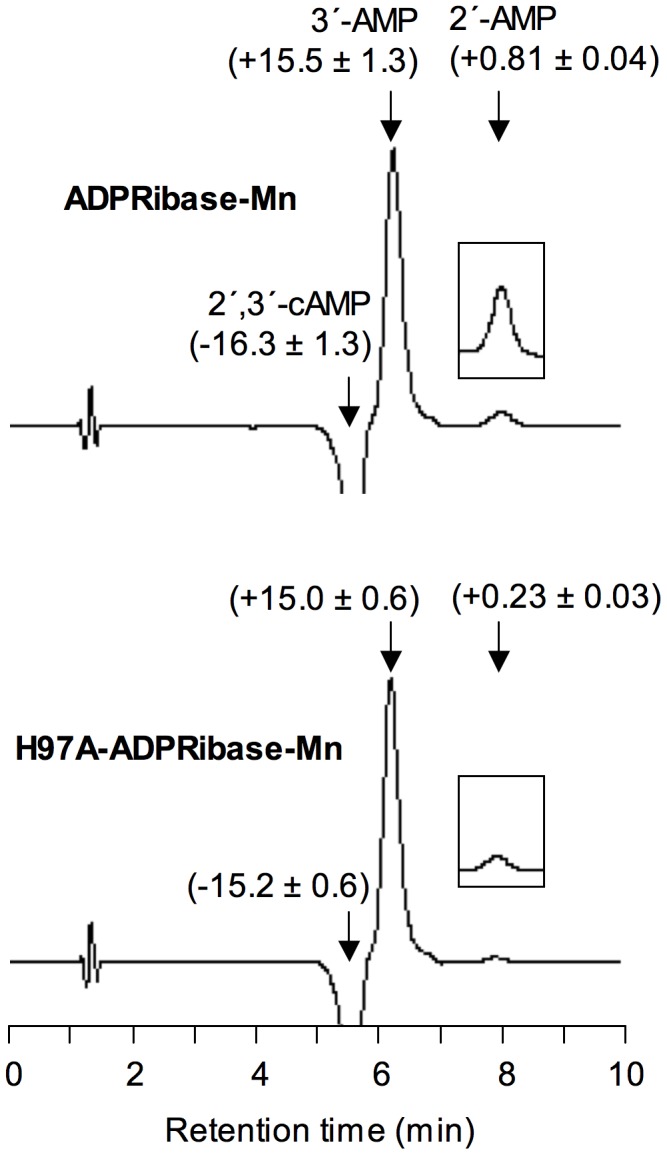
Hydrolysis of 2′,3′-cAMP by zebrafish wild type- and H97A-ADPRibase-Mn. Enzyme reaction mixtures with 500 µM 2′,3′-cAMP and 5 mM MnCl_2_ were incubated for 50 min. Similar unit amounts of wild-type and mutant enzymes were used to facilitate comparison. Each trace is a differential chromatogram showing the difference between samples incubated with enzyme minus no-enzyme controls. The arrows mark the retention times of commercial standards of the indicated compounds. The insert boxes are 5-fold amplifications of the 2′-AMP peaks. The numbers correspond to percent 2′,3′-cAMP consumption or 3′-AMP and 2′-AMP formation (means±S.D. of 3 assays).

A preliminary set of activity assays of wild-type ADPRibase-Mn was performed at a fixed, 0.5 mM concentration of the tested compounds with 5 mM Mn^2+^ as the activating cation. The enzyme hydrolyzed the phosphoanhydride linkages of ADP-ribose, CDP-glycerol, CDP-choline, CDP-ethanolamine and ADP at significant rates, one of the products of hydrolysis being AMP or CMP in every case (HPLC assays). The phosphodiester 2′,3′-cAMP was also attacked at a rate similar to other substrates, and the formation of 3′-AMP as the major product (95%; formed by hydrolysis of the P-O2′ linkage) together with a minor proportion of 2′-AMP (5%; formed by hydrolysis of the P-O3′ linkage) was observed by HPLC (Fig. 2 upper panel). On the contrary, cADPR, which is a known substrate of the rat enzyme, was hydrolyzed by zebrafish ADPRibase-Mn only at a very low, marginal rate. Anyhow, HPLC assays showed the formation of *N* ^1^-(5-phosphoribosyl)-AMP as the product of cADPR phosphohydrolysis like with the rat enzyme [12]. Not attacked at detectable rates were ADP-glucose, UDP-glucose, CDP-glucose, CDP, CMP, AMP, and 3′,5′-cAMP. Since so far the cyclic 3′,5′- and 2′,3′-phosphodiester nucleotides had not been tested as potential substrates of rat ADPRibase-Mn, we did it now and found them to behave as with the zebrafish enzyme.

The results of kinetic studies of zebrafish ADPRibase-Mn with different substrates are summarized in Table 1, where they are compared with the data obtained for the point mutant, H97A-ADPRibase-Mn, and with the rat enzyme. For the wild-type zebrafish enzyme, the highest *k*_cat_ values were for the hydrolysis of CDP-alcohols. However, ADP-ribose was the best substrate, as judged from the higher catalytic efficiency (*k*_cat_/*K*_M_), which depended on a very favorable *K*_M_ value, 15–240 fold lower than those of the other significant substrates.

**Table 1:** Substrate specificity of ADPRibase-Mn enzymes.

Substrate	Zebrafish wild type^a^			Zebrafish H97A mutant			Rata,b
	*K*_M_	*k*_cat_/*K*_M_	*k*_cat_	*K*_M_	*k*_cat_/*K*_M_	*k*_cat_ s^−1^	*k*_cat_/*K*_M_
	µM	M^−1^s^−1^	s^−1^	µM	M^−1^s^−1^	s^−1^	M^−1^s^−1^
ADP-ribose	53±11	(3.4±0.01)×10^5^	18.0	71±8	(5.9±0.5)×10^3^	0.4	3.3×10^5^
CDP-glycerol	2000±1400	(4.8±0.07)×10^4^	95.6	6300±900	(1.2±0.3)×10^2^	0.8	9.2×10^4^
CDP-ethanolamine	3900±1300	(3.2±0.03)×10^4^	125.2	31000±800	(7.0±2.0)×10^1^	2.1	2.9×10^4^
CDP-choline	12000±3000	(5.6±0.8)×10^3^	67.2	43000±8000	(2.0±0.5)×10^1^	0.8	9.5×10^4^
ADP	2650±1300	(6.6±0.7)×10^2^	1.8	19000±700	(1.1±0.5)×10^1^	0.2	2.1×10^4^
2′,3′-cAMP^c^	760±50	(2.2±0.3)×10^4^	16.9	1700±240	(1.8±0.1)×10^4^	29.7	–
cADPR	190±25^d^	(2.8±0.3)×10^1^	0.005	nd^e^	nd	nd	4.2×10^3^

*K*_M_ and *k*_cat_/*K*_M_ values for zebrafish wild-type and mutant enzyme are shown as means±S.D. (*n* = 3–6), and *k*_cat_ values are derived from them.

a Neither the zebrafish wild type nor the rat enzyme display significant activity towards ADP-glucose, UDP-glucose, CDP-glucose, CDP, CMP, AMP or 3′,5′-cAMP.

b Data taken from [11], [12].

c The kinetic parameters of 2′,3′-cAMP hydrolysis correspond to the total activity, including the 3′-AMP-forming hydrolysis of the P-O2′ and the 2′-AMP-forming hydrolysis of the P-O3′ linkage. However, given the strong predominance of the P-O2′ linkage hydrolysis, the parameters may be reasonable approximations to this reaction kinetics.

d Estimated from the potency of (competitive) inhibition of ADP-ribose hydrolysis by cADPR.

e nd, not determined.

The saturation kinetics of the cADPR phosphohydrolase activity was not directly studied due to the very low activity. However, since the docking simulations of substrate binding to the active center (see below) predicted that cADPR could be a competitive inhibitor of other ADPRibase-Mn reactions, its effect on the hydrolysis of ADP-ribose was tested under conditions in which the hydrolysis of cADPR itself was negligible. cADPR was an effective inhibitor of ADP-ribose hydrolysis: 160 µM cADPR inhibited the initial rate of hydrolysis of 80 µM ADP-ribose by 25±2% (*n* = 3). This, assuming the inhibition was competitive, and taking into account the 53 µM *K*_M_ for ADP-ribose as the substrate, pointed to a 190 µM *K*_M_ value for cADPR as calculated using equation II. This is within the range of *K*_M_ values obtained (Table 1), only 3–4-fold higher than the *K*_M_ value for ADP-ribose and several-fold lower than the *K*_M_ values for other substrates. On the other hand, from repeated assays at 30–60 µM cADPR, the catalytic efficiency for cADPR hydrolysis was estimated at 28 M^−1^s^−1^, much lower than for any other substrate considered. This very inefficient hydrolysis of cADPR is thus related to an extremely low *k*_cat_ value of 0.005 s^−1^ (Table 1).

When the catalytic efficiencies for significant substrates are considered side by side, zebrafish and rat ADPRibase-Mn are similarly and highly efficient hydrolases of ADP-ribose (≈3.3×10^5^ M^−1^s^−1^), their best substrate, both being also similarly 10-fold less efficient with CDP-ethanolamine (≈3.0×10^4^ M^−1^s^−1^). For the rest of their substrates, the two enzymes differed quantitatively: the zebrafish one was 2-fold (CDP-glycerol), 17-fold (CDP-choline), 34-fold (ADP) and 150-fold (cADPR) less efficient than the rat one (Table 1). Therefore, the specificity for ADP-ribose is stricter with the zebrafish than with the rat enzyme. The most remarkable difference between them concerned the phosphohydrolysis of cADPR. Rat ADPRibase-Mn hydrolyzes the phosphoanhydride linkage of cADPR with a catalytic efficiency of 4160 M^−1^s^−1^ [12], compared to the almost negligible efficiency of 28 M^−1^s^−1^ shown by the zebrafish enzyme. This seems a genuine difference between mammalian and fish enzymes, because we have preliminary results that recombinant human ADPRibase-Mn hydrolyzes cADPR with a catalytic efficiency similar to the rat enzyme, about 4000 M^−1^s^−1^ (unpublished data). To further study the significance of the lack of activity of zebrafish ADPRibase-Mn towards cADPR, the *Danio rerio* genome was queried for homologues of the human CD38 or BST-1 cADPR synthetases/glycohydrolases. These Blast searches gave negative results indicating that the known pathways of cADPR synthesis may be absent in zebrafish. If this reflects a general phylogenetic situation in which the occurrence of ADPRibase-Mn enzymes active on cADPR parallels that of synthetic enzymes, it would strengthen the relevance of mammalian ADPRibase-Mn in cADPR signaling.

Concerning the ADPRibase-Mn activity on 2′,3′-cAMP, yielding mainly 3′-AMP, it is worthwhile mentioning that this novel activity may be related to the low activation energy for the hydrolysis of the P-O2′ as compared to the P-O3′ linkage, and it is a common feature of many phosphodiesterases belonging or not to the MDP superfamily. It has been speculated that enzymes forming specifically 2′-AMP must be designed to preclude 2′,3′-cAMP adopting orientations that favor the hydrolysis of the P-O2′ bond [39]. This is not the case of ADPRibase-Mn, as 2′,3′-cAMP docking resulted in poses having two different orientations (see below), each prone to hydrolyze either the P-O2′ or the P-O3′ bond, but the 3′-AMP-forming reaction was strongly dominant. Therefore, the hydrolysis of 2′,3′-cAMP by ADPRibase-Mn seems another manifestation of this ‘non-specific’ activity of phosphodiesterases having either a broad or a strict substrate specificity.

The H97A mutant of the zebrafish enzyme showed heavy losses of catalytic efficiency with respect to the wild type with most of the susbtrates: 60-fold decrease over ADP-ribose and ADP; 300–500 fold over CDP-alcohols. This was mainly related to marked decreases of *k*_cat_ (45–120 fold) and relatively minor increases of *K*_M_ (1.3–8 fold) with the only exception of ADP which displayed changes of similar magnitude (but different sign) in both parameters. Particularly worth of note was the very small effect of the mutation on the *K*_M_ for ADP-ribose which increased only 1.3-fold (Table 1).

Unexpectedly, among all those changes caused by the H97A mutation, the catalytic efficiency observed with 2′,3′-cAMP as the substrate was practically untouched as a 2-fold increase of *K*_M_ was almost compensated by an increase of *k*_cat_. In fact, for H97A-ADPRibase-Mn, 2′,3′-cAMP was a better substrate than ADP-ribose (Table 1). Nonetheless, it must be stressed that, although both the total hydrolytic activity (substrate consumption; Table 1) and the strongly predominant P-O2′ hydrolytic reaction (3′-AMP formation; Fig. 2) were little affected by the mutation, the minor P-O3′ hydrolytic reaction (2′-AMP formation) was significantly diminished (Fig. 2 lower versus upper panel).

The small effect of the H97A mutation over the hydrolysis of the P-O2′ linkage of 2′,3′-cAMP could suggest that it depends on an enzyme different than ADPRibase-Mn. To test this possibility, the inhibition by ADP-ribose of the activity of H97A-ADPRibase-Mn towards 2′,3′-cAMP was assayed under conditions of negligible hydrolysis of ADP-ribose. Competition between substrates of a single active site would predict that 2′,3′-cAMP hydrolysis should be competitively inhibited by ADP-ribose with a *K*_i_ equal to its *K*_M_ value. The results agreed with this prediction: 72 µM and 144 µM ADP-ribose inhibited the rate of hydrolysis of 1680 µM 2′,3′-cAMP by 30% and 47%, respectively, while from the competing-substrate theory, using equation II, one would expect 34% and 51% inhibition.

### Mn^2+^ Requirement for Zebrafish ADPRibase-Mn Activity: a Complex Behavior that Indicates the Existence of Multiple Metal Binding Sites

**Figure 3:**
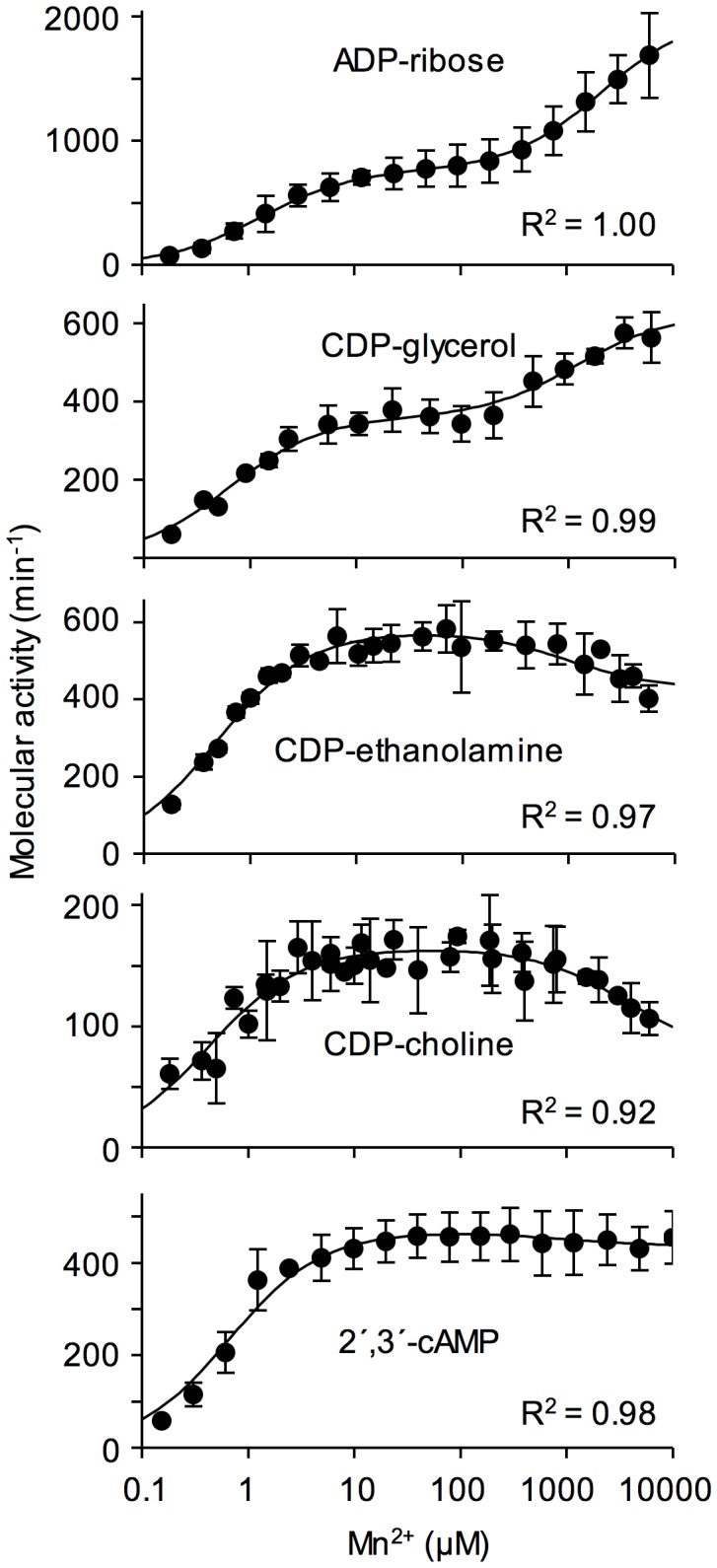
Kinetic response of zebrafish ADPRibase-Mn to changes of Mn^2+^ concentration with different substrates. Rates of substrate hydrolysis were measured in standard reaction mixtures except that Mn^2+^ concentration was varied as indicated. The curves show the best fits of equation I to data points. Error bars correspond to mean±S.D. (*n* = 3). The goodness of the fit is given for each adjustment in terms of R^2^.

**Figure 4:**
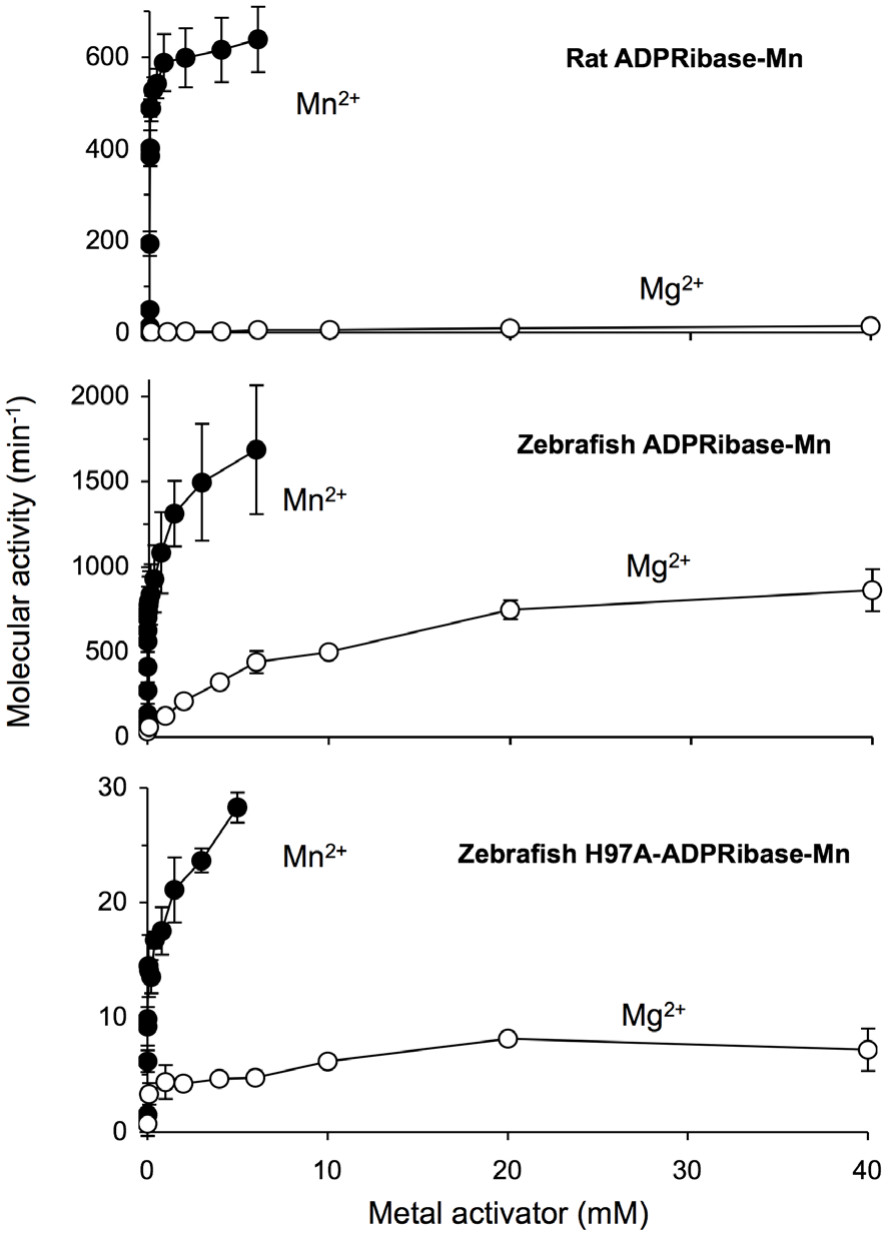
Comparative responses of ADPRibase-Mn enzymes to Mn^2+^ and Mg^2+^ as activators. Rates of substrate hydrolysis were measured in standard reaction mixtures except that Mn^2+^ concentration was varied as indicated, or that Mg^2+^ substituted for Mn^2+^. Error bars correspond to mean±S.D. (*n* = 3).

MDP proteins contain a dimetal center with diverse ion combinations in which, in general, one position can be occupied by Fe^3+^, Mn^2+^, Ni^2+^ or Zn^2+^, and the other by Fe^2+^, Zn^2+^ or Mn^2+^ [3]. Many MDPs require added divalent cations for activity (mainly Mn^2+^, Zn^2+^ or Ni^2+^) which could be due to the loss of native metals during purification, although it has been also argued that in some cases the dinuclear center may contain only one metal in the resting state, and the second one would enter the active site with or upon substrate binding [40]. In fact, the identity of ions in native MDPs is often uncertain, partly because of possible changes during manipulation. For instance, the Mn^2+^-dependent YfcE protein from *E. coli* contains two Zn^2+^ ions when crystallyzed, but they probably bound the protein during purification and reflect a di-Mn^2+^ center [41]. Similarly, zebrafish ADPRibase-Mn has been modeled with two Zn^2+^ ions in the dinuclear center [9], while metal analysis of the recombinant protein before crystallization shows 1.3 mol Fe and 0.23 mol Mn per mole of protein [7]. In our hands, the recombinant enzyme was dependent on Mn^2+^ for full activity (Figs. 3 and 4), like rat ADPRibase-Mn purified from liver or expressed in bacteria [10], [11]. Also, inhibition of the rat enzyme by chelating agents, points to a strongly bound ion other that Mn^2+^ [11]. Altogether, these results support the view that active ADPRibase-Mn contains a Fe-Mn heteronuclear center as suggested by Bitto *et al*. [7]. The Fe ion, either Fe^2+^ or Fe^3+^, would remain firmly bound in the purified enzyme, while Mn^2+^ would be lost and should be exogenously added to reconstitute the Fe/Mn center.

Concerning the response to the addition of exogenous Mn^2+^, rat ADPRibase-Mn activity shows a near hyperbolic dependence on Mn^2+^ reaching saturation around 20 µM with an essentially constant activity at higher Mn^2+^ concentrations up to at least 5 mM (see Fig. 4 upper panel). Since saturation occurs with Mn^2+^ well below substrate concentration used in the enzyme assay (typically set at 500 µM), it is likely that under this condition Mn^2+^ acts by binding to the enzyme, and the dimetal center is fully occupied [10], [11].

In contrast, the zebrafish enzyme displayed a more complex behavior, as its apparent metal saturation curve for ADP-ribose hydrolysis was biphasic when Mn^2+^ concentration was increased from low micromolar to 6 mM. In a preliminary analysis, this curve fit well the sum of two Michaelis-Menten equations with apparent *K*_M_ values of around 1 µM and 2.5 mM Mn^2+^. A similar response to Mn^2+^ was obtained with CDP-glycerol as the substrate. However, this was not so with CDP-choline, CDP-ethanolamine or 2′,3′-cAMP. With these substrates, only the lower apparent *K*_M_ was observed and, after reaching a plateau of ADPRibase-Mn activity between 10 µM and 1 mM Mn^2+^, higher metal concentrations were inhibitory, although with 2′,3′-cAMP such inhibition was almost negligible and the apparent saturation curve was nearly hyperbolic. This complex response to increasing Mn^2+^ concentrations with different substrates cannot be explained simply by the combined action of two independent enzyme forms, one with high and the other with low metal affinity. Instead, the results are interpretable in terms of two Mn^2+^ binding sites that affect differentially the activities on different substrates. A high-affinity Mn^2+^-binding event would be required for activity on all the substrates, and it would correspond to the occupation of the Mn^2+^ site of the dinuclear center. A low affinity Mn^2+^-binding event would be activatory for ADP-ribose and CDP-glycerol, inhibitory for CDP-choline or CDP-ethanolamine, almost without effect for 2′,3′-cAMP and it would correspond to the occupation of an allosteric Mn^2+^ site(s) outside the dinuclear center. A possible location for this external site is pointed out by the crystallographic data [9] (see below).

From the kinetic point of view, the responses of zebrafish ADPRibase-Mn can be described for instance by the scheme shown in Fig. 1, which includes independent metal (X) binding to two different enzyme sites with high and low affinities. These binding events form EX and XE complexes, respectively. EX can bind substrate to form the active EXS complex that yields products with a rate constant *k*. XE does not bind substrate directly but it can do it after binding a second metal ion to form the complex XEXS, which is also able to yield products with rate constant *k–*. For ADP-ribose and CDP-glycerol, *k*′> *k*; for CDP-choline and CDP-ethanolamine, *k*′ <*k*; and for 2′,3′-cAMP, *k*′≈*k*. Such reaction scheme (Fig. 1) responds to equation I, where *K*_A_ and *K*_B_ are the dissociation constants for EX and XE, respectively.

Equation I was adjusted with satisfactory results to all the datasets obtained with increasing Mn^2+^ concentrations and different substrates at a fixed concentration (Fig. 3). Separate adjustments were run for each substrate dataset leaving six fluctuating variables (*V*, *V′*, *K_S_*, *K′_S_*, *K_A_* and *K_B_*), with the only imposed restrictions of avoiding negative values for those parameters and of keeping the substrate *K*_M_ calculated from the fit equation equal to its experimental estimate (Table 1). From the reaction scheme of Fig. 1 one would expect that *K_A_* and *K_B_* were independent on the substrate used. Nevertheless, the errors of the estimates of these values were very large due to the number of fluctuating variables (such that similar adjustments could be obtained with different sets of constant values), to the absence of data obtained at Mn^2+^ concentrations higher than 10 mM (which makes *V′* estimation imprecise), and to the possibility that low total Mn^2+^ concentrations differ significantly from free ion levels (which makes *K_A_* estimation imprecise). Despite these limitations, it is worth considering that, after best fit to the different substrate datasets, *K*_A_ adopted always a micromolar value (0.4–1.0 µM; except 17 µM with ADP-ribose, which could indicate a higher chelation of Mn^2+^ by this substrate compared to the others), while *K*_B_ adopted a millimolar value in every case (1.0–3.6 mM). Overall, this means that the kinetic results agreed with the reaction scheme of Fig. 1.

The high-affinity Mn^2+^-binding to zebrafish ADPRibase-Mn, which had similar activatory effects with all the substrates, seems equivalent to the Mn^2+^ activation of the rat enzyme. The activity plateau reached in the 5–100 µM Mn^2+^ range, at 500 µM substrate, is likely to reflect also full occupancy of the dimetal center of the zebrafish enzyme. It follows that the low-affinity Mn^2+^-binding step should be ascribed to a different site(s). This is also supported by the response of H97A-ADPRibase-Mn to Mn^2+^, studied with ADP-ribose as the substrate (Fig. 4). The results showed strongly diminished activity as expected from the mutant kinetic parameters shown in Table 1, but the apparent metal-saturation curve of the mutant enzyme was biphasic like the one shown by the wild-type enzyme (Fig. 4, lower *versus* central panel). Adjustment of the data to equation I gave values of *K_A_*≈130 µM (i.e. 8-fold higher than for the wild-type enzyme) and *K_B_*≈1 mM (i.e. similar to the wild-type enzyme). The detrimental effect of the H97A mutation on the high-affinity binding of Mn^2+^ is in agreement with its relationship to the dimetal center, as although His-97 is not coordinated to it, its neighbor Asn-96 is [7], [9]. On the other hand, the lack of effect of the mutation on the low-affinity site, supports that it reflects binding to a different site(s). Interestingly, zebrafish ADPRibase-Mn crystals contain two metal ions with low occupancy bound to an external domain, far apart from His-97 and properly placed to have regulatory effects [9] (see below), something not previously postulated for other MDPs.

### Partial Activation of Wild-type and H97A Mutant ADPRibase-Mn by Mg^2+^

**Figure 5:**
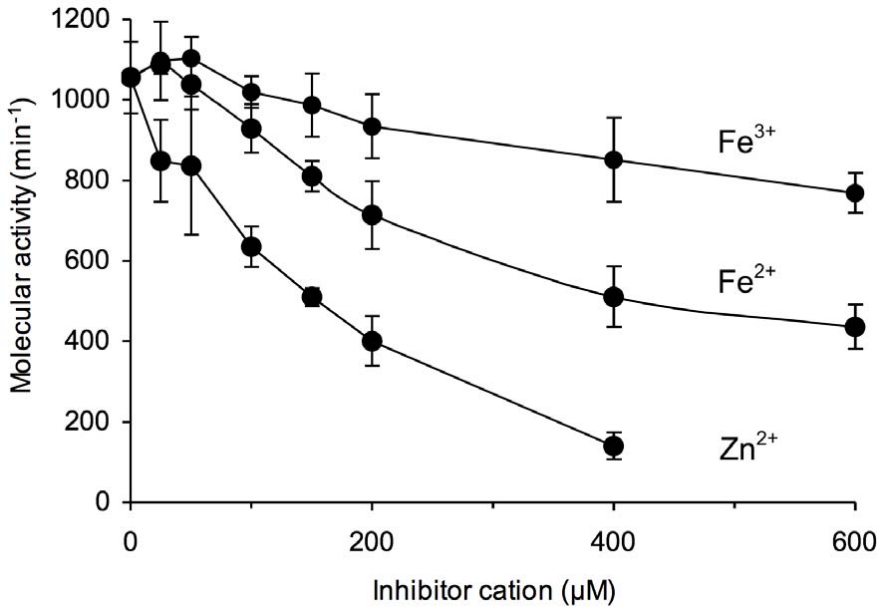
Inhibition of the Mn^2+^-dependent activity of zebrafish ADPRibase-Mn by Zn^2+^, Fe^2+^ and Fe^3+^. Rates of substrate hydrolysis were measured in standard reaction mixtures except that Mn^2+^ concentration was fixed at 30 µM, and the indicated concentrations of an inhibitor cation was added in the form of chloride salts. Error bars correspond to mean±S.D. (*n* = 3).

Zn^2+^, Fe^2+^, Fe^3+^ and Mg^2+^ were tested as metal activators of zebrafish ADPRibase-Mn besides Mn^2+^. Of those, only Mg^2+^ gave positive results (Fig. 4) and, in fact, when Zn^2+^, Fe^2+^, and Fe^3+^ were tested over Mn^2+^-activated ADPRibase-Mn, they behaved as inhibitors (Fig. 5). This effect of Fe ions is not in contradiction with a Fe/Mn dimetal center being required for ADPRibase-Mn activity. The required Fe ion would be firmly bound to the purified protein (see above and [7]). Therefore, the presence of exogenous Fe ions in the assay mixtures to which Mn^2+^ is added does nothing but interfere with the required binding of Mn^2+^ to complete the dimetal center.

Enzymes activated by Mn^2+^ are often activated also by Mg^2+^, which in many cases is the physiological activator. This is not the case for rat ADPRibase-Mn, as it is essentially inactive with Mg^2+^ [10], [11] (see also Fig. 4). However, when the zebrafish enzyme activity on ADP-ribose was tested for Mg^2+^-dependent activation, a significant response was observed, even if much smaller than that evoked by Mn^2+^ and without clear evidence of a biphasic response like the one observed with Mn^2+^ (Fig. 4).

As the activation by Mg^2+^ was one of the differences found between the rat and the zebrafish enzymes, we decided to test whether it was a genuine one, or the Mg^2+^-dependent activity of zebrafish ADPRibase-Mn could represent contamination by another hydrolase, perhaps from the *E. coli* cells used for expression. To clarify this matter, H97A-ADPRibase-Mn was tested for activation by Mn^2+^ or Mg^2+^. It was expected that the H97A mutation would affect the Mg^2+^-dependent activity only if it belonged to the Mn^2+^-dependent ADPRibase-Mn. The results of Fig. 4 show that the mutation diminished both the Mn^2+^- and the Mg^2+^-dependent activity on ADP-ribose to the same extent. This proved the genuineness of the activation of zebrafish ADPRibase-Mn by Mg^2+^.

### pH Dependence of ADPRibase-Mn Activities

**Figure 6:**
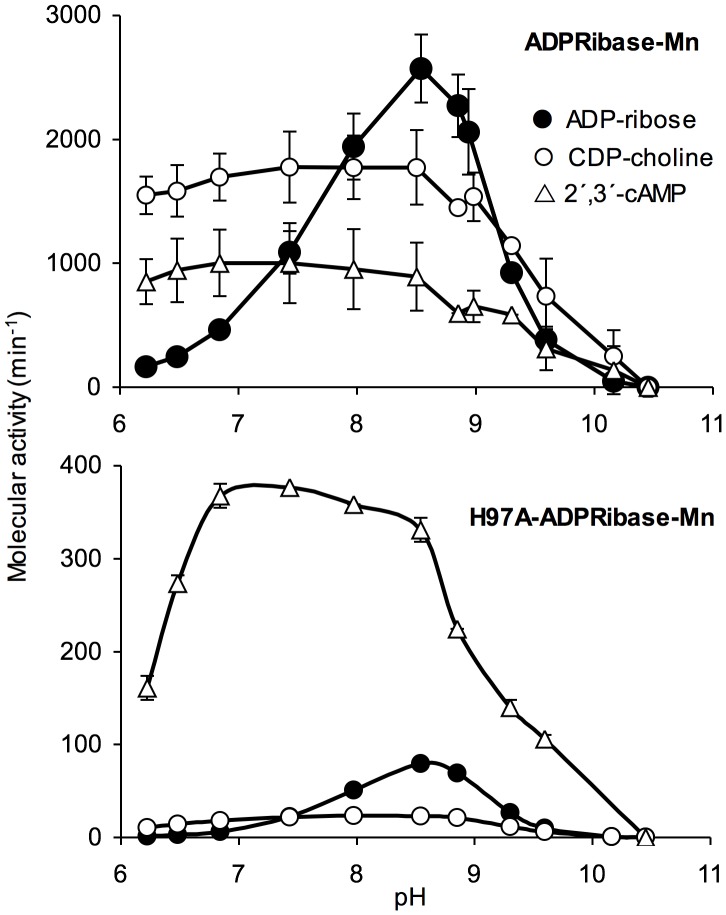
pH-activity profiles of zebrafish ADPRibase-Mn. Rates of substrate hydrolysis were measured in standard reaction mixtures except that different pH buffers were used: Tris/acetate (pH 6.0–7.0), Tris-HCl (pH 7.5–9.5), glycine/NaOH (pH 9.8–10.8). The pH values shown in the plots were measured in the reaction mixtures. Error bars correspond to mean±S.D. (*n* = 3).

Fig. 6 shows the pH-activity profiles of ADPRibase-Mn and H97A-ADPRibase-Mn with different substrates. The profile obtained with ADP-ribose was bell shaped, with an optimum for activity near pH 8.5, and half-maximum activities around pH 7.6 and pH 9.2. The right-hand side moieties of the profiles observed with CDP-choline and 2′,3′-cAMP (above pH 8.5) were similarly shaped, but below pH 8.5 the activities on these substrates remained practically constant until near pH 6, when the activity on ADP-ribose was negligible. The mutant H97A-ADPRibase-Mn showed similar profiles to the wild-type enzyme but with the already mentioned strong losses of activity over ADP-ribose and CDP-choline.

The substrate-dependent pH profiles may indicate that a functional group that ionizes around pH 7.6 is required in the unprotonated form for the enzyme to be active on ADP-ribose, but not on CDP-choline and 2′,3′-cAMP. However, this kind of reasoning would require a more detailed study of the ADPRibase-Mn response to pH, including *k*_cat_, *k*_cat_/*K*_M_ and *K*_M_ profiles for all the substrates. On the other hand, the identification of such a functional group will require also further study.

### Positioning of Substrates in the Active Site by Docking Simulation: Orientation with Respect to His-97, and to a Water Molecule Bridging the Dimetal Center

Current evidence indicate that MDP enzymes act through a direct nucleophilic attack by a water molecule (or hydroxide) coordinated to the dimetal center, without the formation of covalent enzyme-product intermediates [3]. However both the precise nucleophile and the substrate binding mode are controversial. Two different proposals assume the nucleophile is either (i) terminally bound to one of the metal ions [1], [40] or (ii) bound in a bidentate fashion to both metal ions [42], [43]. In the first case the substrate must bind terminally to one of the metal ions while the nucleophile is bound to the other. In the second, the substrate could bind to the dimetal center through the formation of a μ-phosphate bridge. The crystallographic structure of zebrafish ADPRibase-Mn contains P_i_ bidentately bound to the two ions of the dimetal center and a bridging molecule of water also bidentately bound to the metal ions [7], [9]. This and the remarkable specificity of zebrafish ADPRibase-Mn prompted us to perform simulations of substrate docking to the active site, so as to explore how the dimetal center, its bridging water and amino acid residues of the active site could interact with ADPRibase-Mn substrates. Before attempting to dock substrate molecules to the active site, we considered two aspects of the active site: the bridging water and the identity of the dinuclear center ions.

According to data in the PDB file, the bridging water shows a 100% occupancy and a B factor or temperature factor much lower (≈20 Å^2^) than other molecules of water present in the vicinity (≥35 Å^2^), and almost two standard deviations below the mean B factor of the ≈350 molecules of water present in the crystal (39.9±10.8 Å^2^). This means that the bridging water appears well defined with a high confidence in its crystal position. Anyhow, the effect of its presence was tested by running P_i_ docking experiments to the protein model without the bridging water or including it as either the neutral molecule or a hydroxide ion. These simulations indicated that the best reproduction of the crystallographic position of P_i_ was obtained with a bridging, neutral molecule of water. Regarding the metal ions, we considered that, although a dinuclear center with two Zn^2+^ ions, as it appears in the protein crystals, does not possibly correspond to the active form of ADPRibase-Mn, there would be a large degree of uncertainty in the substitution of a Fe/Mn center for the di-Zn one. The uncertainty would be related to the oxidation state and position occupied by the Fe ion in the heteronuclear center, and to any effects on the conformation of relevant amino acid residues of the active center. Therefore, the structure used to simulate substrate docking contained the bridging water molecule in the neutral state, and kept the di-Zn nature of the dinuclear center. One should be aware that these decisions may have some influence on the results.

**Figure 7:**
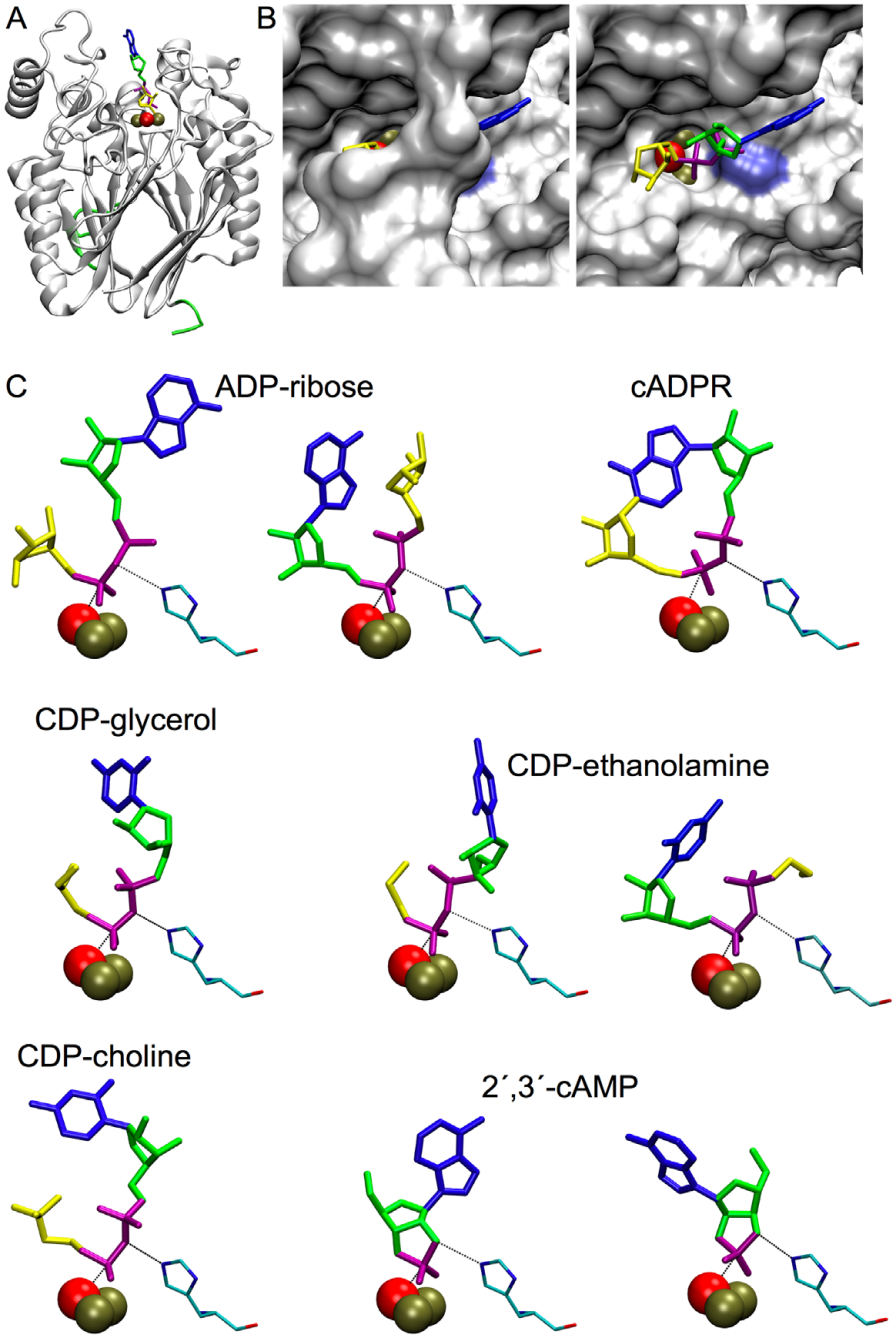
Substrates docked to the crystal structure of zebrafish ADPRibase-Mn. (A) General view with ADP-ribose docked to the active site like in the first individual pose shown in C. (B) Upper view of A showing (left) the full protein surface and (right) a view with part of the protein removed to allow vision of bound ADP-ribose. (C) Series of poses found for the different subtrates by docking. For ADP-ribose, CDP-ethanolamine and 2′,3′-cAMP, two poses with different orientations are shown. The histidine residue of the GNH[D/E] motif, His-97, is shown in every pose. Bronze spheres, metal ions of the dinuclear center; red sphere, bridging molecule of water, which is assumed to act as nucleophile in the ADPRibase-Mn reactions. The figures were prepared with the Visual Molecular Dynamics (VMD) program [50].

Fig. 7 shows a selection of the docking images obtained with ADP-ribose, cADPR, CDP-glycerol, CDP-ethanolamine, CDP-choline and 2′,3′-cAMP, which highlight the spatial relationship of the studied compounds with His-97, and with the bridging water molecule. The position of one of the substrate P atoms was always very near (0.2–0.7 Å, except for cADPR 1.2 Å) to that of the P_i_ bound to the protein crystal structure and, with the exception of cADPR, the corresponding phosphate group was in a reasonable position to form, like P_i_ in the crystal, a bidentate complex with the dimetal center. The bridging water molecule was within a distance adequate to perform a nucleophilic attack on that P atom. The water nucleophile stayed near in line with the scissile P-O bond in all the poses shown (168.2°–174.8°), again with the relevant exception of cADPR (129.5°). Although easily docked into the active site, cADPR could not adopt a pose susceptible of being attacked in line, which is in agreement with a *K*_m_ for cADPR not much higher than that for ADP-ribose, but with an extremely low *k*_cat_ for cADPR hydrolysis, as found by experiment.

Among the ADPRibase-Mn substrates docked, for ADP-ribose, CDP-ethanolamine and 2′,3′-cAMP, similarly favorable poses were found which showed opposite ligand orientations. For the two former substrates, in each binding orientation, a different P atom was placed in position for nucleophilic attack. This indicated that either AMP (CMP) or phosphoribose (phosphoethanolamine) can act as the leaving group, but the reaction products would be the same in both situations. For 2′,3′-cAMP, the poses presented the single P atom at attack distance by water, but with different P-O bonds in the near in-line position: either P-O2′ or P-O3′. The observation that ADPRibase-Mn hydrolyzed 2′,3′-cAMP, rendering 95% of 3′-AMP and 5% of 2′-AMP as alternative products (Fig. 2), shows that this substrate can effectively bind to the enzyme in two opposite orientations, as indicated by the docking simulations (Fig. 7C). On the contrary, evidence is not at hand to elucidate whether the docking of ADP-ribose and CDP-ethanolamine in two orientations reflects the actual behavior of the enzyme.

In all the ADPRibase-Mn models containing substrates bound to the active site, His-97 (i.e. the GNH[D/E] histidine; see above) was within hydrogen bonding distance of the O-atom participating in the scissile P-O linkage (Fig. 7). The effects of the H97A mutation strongly support a role for this residue in catalysis (Table 1). In other superfamily members, the GNH[D/E] histidine is required for activity although its exact role is not clear [3]. It has been proposed that it could work on the alcohol leaving group either as a catalytic general acid or contributing to the neutralization of its negative charge in the transition state [44]. In ADPRibase-Mn, as long as the enzyme acts as a pyrophosphohydrolase, such a role is potentially less important, because the leaving group is an esterified phosphate like AMP (or phosphoribose) in the case of ADP-ribose hydrolysis. Only in the case of 2′,3′-cAMP hydrolysis there is an alcohol leaving group but then, the H97A mutation had little effect on the *k*_cat_ value for this reaction (Table 1). An interesting alternative possibility is that, like in the purple acid phosphatases family [45], the role of the GNH[D/E] histidine is mainly in substrate positioning. The strong decrease of the *k*_cat_ values of phosphoanhydride hydrolysis due to the H97A mutation (Table 1) would thus be explained by the lesser probability for the substrate to adopt the in-line orientation without the help of His-97. The marked independence of 2′,3′-cAMP hydrolysis to 3′-AMP on this amino acid would then indicate that this substrate adopts easily the correct orientation in the active center, which may be related to its marked rigidity as compared to the other ADPRibase-Mn substrates. In fact, this was supported by careful consideration of the results of the docking experiments.

The binding poses found by docking simulations are not unique for any given ligand; rather they can be grouped into clusters. Therefore the poses shown in Fig. 7C can be considered as representatives chosen each within a cluster composed by members which occupy closely related positions. Among the poses found within the active center in the docking process (100–200 poses depending on the ligand), the Jarvis-Patrick algorithm [31] defined many different clusters: 80–145 for ADP-ribose or each CDP-alcohol, 18 for cADPR, but only 4 for 2′,3′-cAMP, despite the latter being the ligand that gave the largest number of poses within the active center. This indicates a much lesser diversity in the positions potentially adopted by 2′,3′-cAMP than in those adopted by other substrates within the active center. Such a difference could be related to the differential effect of the H97A mutation of zebrafish ADPRibase-Mn on the activities towards 2′,3′-cAMP and the other substrates. For substrates that can adopt many different positions, His-97 could play a role helping to find the productive one. The much restricted diversity of 2′,3′-cAMP positioning in the active site, implies that, once this substrate is within the active center, there are few position choices different to the productive one, thus making His-97 relatively unimportant for 2′,3′-cAMP positioning and hydrolysis.

### About the Proposed Role of the GNH[D/E] Histidine of Metallo-dependent Phosphatases as a Determinant of Activity on 2′,3-cAMP

A study of the 2′,3′-cyclic phosphodiesterase activity of MDPs has emphasized a specific role of the GNH[D/E] histidine in such activity by comparing the behavior of wild-type and mutant forms of three different dinuclear phosphohydrolases [33]. *Clostridium thermocellum* polynucleotide kinase/phosphatase (*Cth*Pnkp), is a monophosphatase/phosphodiesterase, which among other things hydrolyzes 2′,3′-cAMP rendering exclusively 2′-AMP. Mutation of the GNH[D/E] histidine of *Cth*Pnkp (His-264 in this case) to Ala, Asn or Gln renders a mutant enzyme with much lower activity on 2′,3′-cAMP, but higher activity on bis-*p*-nitrophenylphosphate [46]. *Mycobacterium tuberculosis* Rv0805 is an enzyme first described as 3′,5′-cNMP phosphodiesterase [47], but later shown to be 150-fold more active on 2′,3′-cAMP, yielding mainly 3′AMP but also a minor amount of 2′-AMP. Mutation of the GNH[D/E] histidine of Rv0805 (His-98 in this case) to Ala or Asn suppressed the 2′,3′-phosphodiesterase activity without affecting activity on bis-*p*-nitrophenylphosphate [33]. *E. coli* YfcE hydrolyzes artificial phosphodiester substrates, but not, or only very slowly, 2′,3′-cNMP [41]. In this enzyme, a histidine residue equivalent to those of the previous enzymes is absent and replaced by Cys-74. When this cysteine is mutated to histidine, the modified YfcE protein acquires a vigorous 2′,3′-cNMP phosphodiesterase activity that yields only 3′-NMP as the product [33]. Based on these results, it has been pointed out that the GNH[D/E] histidine is a determinant of 2′,3′-cyclic nucleotide phosphodiesterase activity [33]. However, the fact that zebrafish ADPRibase-Mn mutation at His-97, which is the GNH[D/E] histidine in this protein, was strongly detrimental for activities on all substrates except 2′,3′-cAMP does not fit into that generalization. Things must be more complicated and the activity of MDPs on 2′,3′-cNMP cannot be attributed solely to a conserved GNH[D/E] histidine residue, but is probably related to additional factors acting either positively or negatively.

### Structural Elements Unique to the ADPRibase-Mn-like Family within the Metallo-dependent Phosphatase Superfamily

**Figure 8:**
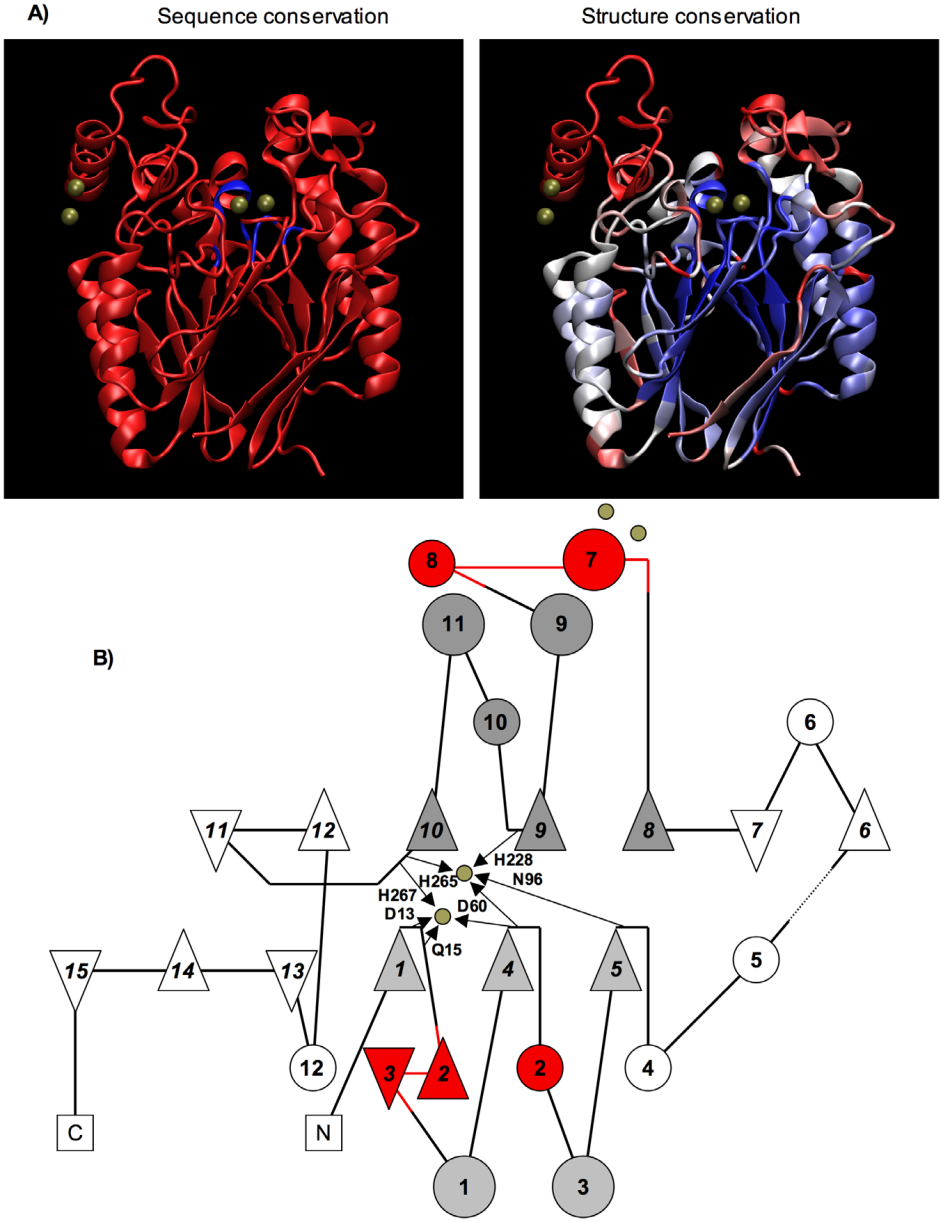
Structural elements unique to the ADPRibase-Mn-like family within the MDP superfamily. (A) Conservation of sequence and structure of prototypical zebrafish ADPRibase-Mn (PDB ID 2nxf) within the SCOP MDP superfamily. From the 44 structural homologues returned by a DALI search (Fig. S1), a group of 14 structures was selected (Table S1), including the closest structural homologue and members of the other 11 families of the superfamily. A structural alignment of 2nxf and these 14 homologues was generated with the VMD MultiSeq plugin [51], and it was used to color zebrafish ADPRibase-Mn by sequence or structure conservation (blue, conserved; red, not conserved). Bronze spheres, metal ions. (B) Topology diagram of zebrafish ADPRibase-Mn according to structural elements summarized in PDBSum (http://www.ebi.ac.uk/pdbsum; triangle, β strand; circle, α-helix, except 6, 8, 10 and 12 which are 3_10_ helices). The two βαβαβ motives are shaded in different gray tones. Parts shaded in red are the unique elements of zebrafish ADPRibase-Mn delimiting the active center entrance. Bronze circles, metal ions; small arrows and names, amino acids coordinated to metals in the dinuclear center.

ADPRibase-Mn-like proteins are classified by SCOP as a unique family within the MDP superfamily. Like the other proteins of the superfamily, zebrafish ADPRibase-Mn contains a 4-layer α/β/β/α fold (SCOP ID 56299), but the two βαβαβ motifs that form it are interrupted by additional elements (Fig. 8). To find out what the unique structural aspects of these proteins could be, a search for structural homologues of zebrafish ADPRibase-Mn was run in the DALI database (http://ekhidna.biocenter.helsinki.fi/dali) [48] against the PDB90 subset of the Protein Data Bank (PDB). The search returned 44 matches that are shown structurally aligned to zebrafish ADPRibase-Mn in Fig. S1. From these, a set of proteins covering all the other families of the SCOP MDP superfamily was chosen for further analysis (Table S1). Against this background, zebrafish ADPRibase-Mn showed very little sequence conservation, but a high degree of structure conservation (Fig. 8A). Only a few protein parts of ADPRibase-Mn were structurally not conserved and could be unique to the ADPRibase-Mn-like proteins. Among them, three are regions with (almost) no counterpart in the other superfamily members. One corresponds to amino acids aprox. 20–35; it contains a β-hairpin motif intercalated between the left βα element of the first βαβαβ motif, forming a small independent β sheet (Fig. 8B, strands 2 and 3). Another is formed by amino acids aprox. 65–70 and folds as a small α-helix, which follows the central β element of the same motif (Fig. 8B, helix 2). The third is a domain formed by amino acids aprox. 150–195, which interrupts the second βαβαβ motif and includes a large α-helix where two metal ions different from those of the dinuclear center are bound in the crystal structure with low occupancy (Fig. 8B, helices 7 and 8). Interestingly, all these elements unique to ADPRibase-Mn-like proteins delimit the active site entrance. A BlastP search showed they are conserved in the ADPRibase-Mn orthologues in terms of sequence. They are also conserved in terms of structure in ADPRibase-Mn proteins that have been modeled by homology to the zebrafish protein (Swiss-Model repository; http://swissmodel.expasy.org/repository/; [49]).

### Recapitulation and Final Remarks

This work demonstrates that the pyrophosphatase activity on both ADP-ribose and CDP-alcohols is indeed a general feature of ADPRibase-Mn-like family members, as it is displayed by the rat ADPRibase-Mn [10], [11] and by its zebrafish orthologue (this work) which is the prototype of the structural family. On the contrary, the unique pyrophosphatase activity on cADPR [12] is essentially restricted to part of the family, as this activity is near negligible with the zebrafish enzyme (this work). This correlates, according to docking simulations, with the inability of cADPR to fit into the active site well positioned for in-line attack by a water nucleophile. A novel activity of ADPRibase-Mn enzymes as 2′,3′-cyclic phosphodiesterases has been detected and characterized. Our results support the hypothesis of Rao *et al.* [39] that 2′,3′-cAMP hydrolysis to 3′-AMP is a general feature of many phosphodiesterases having different physiological substrates, while pointing out that the histidine residue of the GNH[D/E] motif (His97, in the zebrafish enzyme) is not by itself a determinant for phosphodiesterase activity on 2′,3′-cAMP as suggested by others [33]. Characterization of zebrafish H97A-ADPRibase-Mn indicates that the mutated residue is important for catalysis with all the substrates except 2′,3′-cAMP, as in this case the catalytic efficiency was not affected by the mutation.

Three structural elements, specific of this protein family, have been identified and may contain residues responsible for enzyme specificity. For instance, they include Arg-31 and the α-helix formed by amino acids 161–175. According to the crystal structure of zebrafish ADPRibase-Mn [7], [9] and to substrate docking models (this work), Arg-31 has its guanidium group in the vicinity of substrate phosphoryl groups, establishing with them an electrostatic interaction that is likely relevant to mechanism. The 161–175 α-helix of ADPRibase-Mn crystals contains an external binding site for metal ions and it is part of a specific domain well placed to have a regulatory role, which could constitute the basis for the substrate-dependent, low-affinity phase of the response of the zebrafish enzyme to Mn^2+^ not shown by the rat enzyme. Altogether, this should help to select residues for mutagenic studies pursuing a dissection of the substrate binding site of ADPRibase-Mn enzymes with such a peculiar specificity. A study of this kind is currently underway involving human ADPRibase-Mn.

## Supporting Information

Figure S1 **Structural alignment of homologues of zebrafish ADPRibase-Mn.** A search run in the DALI database (http://ekhidna.biocenter.helsinki.fi/dali) against the PDB90 subset of the Protein Data Bank (PDB) returned the 44 matches shown (default run conditions; done on November 30, 2011). The figure is a direct print of the structural alignment provided by the DALI site, and it is published with permission.
(PDF)

Table S1 **Structures representative of the metallo-dependent phosphatase superfamily.** These structures were selected from the DALI search (Fig. S1) to look for the unique structural elements of the ADPRibase-Mn-like family (Fig. 8).
(PDF)
