# Supplementary material for: Characterization of Danio rerio Mn2+-Dependent ADP-Ribose/CDP-Alcohol Diphosphatase, the Structural Prototype of the ADPRibase-Mn-Like Protein Family
Source: PLoS One. 2012 Jul 27;7(7):e42249. doi: 10.1371/journal.pone.0042249 (PMC3407115; doi:10.1371/journal.pone.0042249)
Supplement: Figure S1 — Structural alignment of homologues of zebrafish ADPRibase-Mn. A search run in the DALI database (http://ekhidna.biocenter.helsinki.fi/dali) against the PDB90 subset of the Protein Data Bank (PDB) returned the 44 matches shown (default run conditions; done on November 30, 2011). The figure is a direct print of the structural alignment provided by the DALI site, and it is published with permission. (PDF) [file pone.0042249.s001.pdf]

DaliLite Results: Multiple structural alignment

Each neighbour is shown in the pairwise Dali-alignment to 2nxfA. Inserted segments relative to the top structure are hidden. You can check the 'Expand gaps' option in the summary page to see the complete sequence of the matched proteins. Uppercase means structurally equivalent positions with 2nxfA. Lowercase means insertions relative to 2nxfA. The first part shows the amino acid sequences of the selected neighbours. The second part shows the secondary structure assignments by DSSP (H/h: helix, E/e: strand, L/l: coil). The most frequent amino acid type is coloured in each column.

```
0001 2nxfA [illegible]
0002 2xmoA [illegible]
0003 3d03A [illegible]
0004 1qhwA [illegible]
0005 1warA [illegible]
0006 1uteA [illegible]
0007 3ib7A [illegible]
0008 1xzwB [illegible]
0009 1kbpA [illegible]
0010 2yvtA [illegible]
0011 1uf3A [illegible]
0012 1ii7A [illegible]
0013 2wdeA [illegible]
0014 3qfkA [illegible]
0015 1ho5A [illegible]
0016 2z1aA [illegible]
0017 3c9fB [illegible]
0018 3ivdA [illegible]
0019 1t71A [illegible]
0020 3gveA [illegible]
0021 3jyfA [illegible]
0022 1t70A [illegible]
0023 2cv9A [illegible]
0024 1su1A [illegible]
0025 1s3lA [illegible]
0026 3ck2A [illegible]
0027 2a22B [illegible]
0028 2r17A [illegible]
0029 3e0jA [illegible]
0030 1xm7A [illegible]
0031 2gjuA [illegible]
0032 3floA [illegible]
0033 2kknA [illegible]
0034 1nnwA [illegible]
0035 2z72A [illegible]
0036 2qjcA [illegible]
0037 1s70A [illegible]
0038 1wao1 [illegible]
0039 2dfjA [illegible]
0040 2nppC [illegible]
0041 3icfA [illegible]
0042 1auiA [illegible]
0043 1g5bB [illegible]
0044 1emsA [illegible]
0045 3hkxA [illegible]
```

```
0001 2nxfA [illegible]
0002 2xmoA [illegible]
0003 3d03A [illegible]
0004 1qhwA [illegible]
0005 1warA [illegible]
0006 1uteA [illegible]
0007 3ib7A [illegible]
0008 1xzwB [illegible]
0009 1kbpA [illegible]
0010 2yvtA [illegible]
0011 1uf3A [illegible]
0012 1ii7A [illegible]
0013 2wdeA [illegible]
0014 3qfkA [illegible]
0015 1ho5A [illegible]
0016 2z1aA [illegible]
0017 3c9fB [illegible]
0018 3ivdA [illegible]
0019 1t71A [illegible]
0020 3gveA [illegible]
0021 3jyfA [illegible]
0022 1t70A [illegible]
0023 2cv9A [illegible]
0024 1su1A [illegible]
0025 1s3lA [illegible]
0026 3ck2A [illegible]
0027 2a22B [illegible]
0028 2r17A [illegible]
0029 3e0jA [illegible]
0030 1xm7A [illegible]
0031 2gjuA [illegible]
0032 3floA [illegible]
0033 2kknA [illegible]
0034 1nnwA [illegible]
0035 2z72A [illegible]
0036 2qjcA [illegible]
0037 1s70A [illegible]
0038 1wao1 [illegible]
0039 2dfjA [illegible]
0040 2nppC [illegible]
0041 3icfA [illegible]
0042 1auiA [illegible]
0043 1g5bB [illegible]
0044 1emsA [illegible]
0045 3hkxA [illegible]
```

Please cite: Holm L, Rosenström P (2010) Dali server: conservation mapping in 3D. Nucl. Acids Res. 38, W545-549.

© Structural Genomics Group
Institute of Biotechnology
University of Helsinki
December 2003
