# Supplementary material for: Characterization of Danio rerio Mn2+-Dependent ADP-Ribose/CDP-Alcohol Diphosphatase, the Structural Prototype of the ADPRibase-Mn-Like Protein Family
Source: PLoS One. 2012 Jul 27;7(7):e42249. doi: 10.1371/journal.pone.0042249 (PMC3407115; doi:10.1371/journal.pone.0042249)
Supplement: Table S1 — Structures representative of the metallo-dependent phosphatase superfamily. These structures were selected from the DALI search (Fig. S1) to look for the unique structural elements of the ADPRibase-Mn-like family (Fig. 8). (PDF) [file pone.0042249.s002.pdf]

Table S1. Structures representative of the metallo-dependent phosphatase superfamily.

Protein (PDB ID)	Structural similarity (DALI Z-score)	SCOP metallo-dependent phosphatase family
2xmoA[a]	26.7	–
3d03A	24.2	GpdQ-like
1qhwA[b]	23.3	Purple acid phosphatase-like (Mammalian purple acid phosphatase)
1kbpA[b]	19.5	Purple acid phosphatase-like (Plant purple acid phosphatase, catalytic domain)
1uf3A	17.3	TT1561-like
1ii7A	16.1	DNA double-strand break repair nuclease
1ho5A domain	13.5	5'-nucleotidase (syn. UDP-sugar hydrolase), N-terminal
2cv9A	11.9	TTHA0625-like
1t70A	11.9	DR1281-like
1s3lA	11.6	YfcE-like
1xm7A	9.8	Hypothetical protein aq_1666
1nnwA	9.0	Phosphoesterase-related
1auiA[c]	6.9	Protein serine/threonine phosphatase (Protein phosphatase-2B; PP-2B, calcineurin A subunit)
1g5bB[c]	6.8	Protein serine/threonine phosphatase (λ Ser/Thr protein phosphatase)

These structures were selected from the DALI search (Fig. S1) to look for the unique structural elements of the ADPRibase-Mn-like family (Fig. 8).

[a] This protein is not yet classified in SCOP, but it shows the general characteristics of the metallo-dependent phosphatase superfamily*, and it is the closest structural homologue of zebrafish ADPRibase-Mn (PDB ID 2nxf).

[b, c] Two structures were selected from each of these SCOP families (SCOP domain indicated in parenthesis) because even classified in the same family they display only a 18% identity.

* Kim, Y. G., Jeong, J. H., Ha, N. C. and Kim, K. J. (2011) Structural and functional analysis of the Lmo2642 cyclic nucleotide phosphodiesterase from *Listeria monocytogenes*. Proteins **79**, 1205-1214
